# Automatic Detection of a Student’s Affective States for Intelligent Teaching Systems

**DOI:** 10.3390/brainsci11030331

**Published:** 2021-03-05

**Authors:** Mark H. Myers

**Affiliations:** Department of Anatomy and Neurobiology, University of Tennessee Health Sciences Center, Memphis, TN 38163, USA; mmyers25@uthsc.edu

**Keywords:** a priori, multi-layer perceptron, naive Bayes, antecedent/consequent, human computer interaction, affective states, intelligent tutoring systems

## Abstract

AutoTutor is an automated computer tutor that simulates human tutors and holds conversations with students in natural language. Using data collected from AutoTutor, the following determinations were sought: Can we automatically classify affect states from intelligent teaching systems to aid in the detection of a learner’s emotional state? Using frequency patterns of AutoTutor feedback and assigned user emotion in a series of pairs, can the next pair of feedback/emotion series be predicted? Through a priori data mining approaches, we found dominant frequent item sets that predict the next set of responses. Thirty-four participants provided 200 turns between the student and the AutoTutor. Two series of attributes and emotions were concatenated into one row to create a record of previous and next set of emotions. Feature extraction techniques, such as multilayer-perceptron and naive Bayes, were performed on the dataset to perform classification for affective state labeling. The emotions ‘Flow’ and ‘Frustration’ had the highest classification of all the other emotions when measured against other emotions and their respective attributes. The most common frequent item sets were ‘Flow’ and ‘Confusion’.

## 1. Introduction

Intelligent tutoring systems have been the ultimate goal in remote learning paradigms for several years. Automatic detection of learning states is part of an intelligent system, where the student’s emotion or affective state is tied to a learning session and is updated to the subsequent automated learning response of the student, such as confusion, high interest, etc. [1,2]. When a student is presented with problems or questions, the intelligent tutoring system will present a dialogue in response to the student based on both the answer and reported current affective state. The system may respond to the student in answering a question to the student, correcting student’s errors, or providing hints or pumps (tell me more) to enable the student to answer the question correctly. Throughout this session, affective states are captured, such as through the method known as emote aloud [3].

Other types of emotive capture have involved affect detection through physiological feature capture. In order to improve the accuracy of learning engagement detection, Zhang et al. [4] have collected two aspects of students’ behavior data: face data and mouse interaction. This study focused on eyes, eyebrows, and the mouth area as very important in generating facial expressions, in order to classify if the student was engaged/not engaged. Mouse movement correlated to different levels of student engagement. The experimental result indicated that the recognition rate of the experiment, which used the mouse movement data with the facial labeled data, is higher than just the facial labeled data (94.60% vs. 91.51%).

Enhanced multimodel feature detection has been implemented using various types of sensors (camera, pressure sensor, eye tracker, etc.) to detect learner engagement [5]. Hardy, Wiebe, Grafsgaard, Boyer, and Lester [6] explored trainees’ skin conductance responses to confirm emotional events such as confusion, frustration, happiness and their relationship to learning during a training session. D’Mello et al. [7] developed a multimodal detector combing dialog cues, body language, and facial features to distinguish affective states such as boredom, confusion, frustration, happiness, and neutral states. Additional sensors have been employed beyond this study which involved body posture measurement and facial features [8]. The authors performed affect classification analyses with posture using k-nearest neighbors. This classifier achieved accuracies of 70%, 65%, 74%, and 72% in detecting boredom, confusion, flow, and frustration. For delight, a logistic regression classifier had the best accuracy of 70%. Additionally, affect detection from facial features involved a set of judges to apply the Facial Action Coding System against students involved in active learning environments, whereas a judge had to label an affect state within 20 s. Through this approach, the classification accuracy for delight was the highest (90%), boredom the lowest (60%), and confusion (76%) and frustration (74%) fell in the middle.

The Computer Expression Recognition Toolbox (CERT) is an automated facial expression recognition system that detects facial expressions from a video stream, such as anger, disgust, fear, joy, sadness, surprise, contempt, as well as 30 facial action units [9,10]. FACET is a commercial software for automatic facial expression measurement based on CERT [11], where it estimates facial expressions in terms of the six basic emotions as well as 20 FACS Action Units (AUs). A study utilizing the FACET system, found that the main emotional affect during a learning session revealed that happiness was the most recognized affective state, F(5, 3731) = 87.99, *p* < 0.001, $\eta_{p}^{2}$ = 0.11, followed by anger, sadness, disgust, surprise, and finally fear [12].

Analysis that focused on the changes of emotions throughout a learning session vs. the end state has produced insights into performance. The intelligent tutoring system, Metatutor captured and analyzed 117 college student’s concurrent and self-reported emotions at three time points during a learning session. Analyses revealed negative relationships between increases in boredom and performance. They also found that if confusion persisted over time during learning session, it was detrimental to performance. The outcome of this research found that with emotional feedback, a learning session can be modulated [13]. Another study that considers several physiological inputs beyond Facial Action Units utilizes eye-tracking data to compute attention, electroencephalograph signals to measure changes in alpha and theta wave to determine cognitive load, and finally a wristband to measure (1) blood volume pulse for the duration of a task, (2) mean heart rate for the duration of a task (3) mean electrodermal activation for the duration of a task and (4) mean skin temperature for the duration of a task. The researchers applied k-means to multimodal data to cluster students’ behavioral patterns. In order to predict students’ effort to complete the upcoming task based on their emotions, they applied a combination of Hidden Markov Models (HMMs) and the Viterbi algorithm. This study focuses deeper into the physiological aspect of learning where cognitive load, heart rate, etc. are classified into groups, i.e., Effortless behavior—Observed: high mental workload, high load on memory and low heart rate (HR) (C1), Effortful behavior—Observed: high electrodermal activity (EDA), high emotion, high HR, low load on memory and low mental workload (C3), etc. [14].

The present study focuses on the affective states of (1) anger, (2) boredom, (3) confusion, (4) contempt, (5) curiosity, (6) delight, (7) flow, and (8) frustration [3,12]. Selection of these states was based on previous experimentation, as an example, a student may feel confusion which occurs from either perceived contradiction, anomalies, or from gaps in knowledge from presented learning material which can lead to the frustration affective state [15,16]. Conversely, the student may enter a learning state of curiousness which may subsequently lead to a eureka state, enabling moments of insight during problem solving sessions [17]. These were the states that were the most dominate affective states found while interacting with Autotutor [18]. Using data collected from AutoTutor, the following determinations were sought: Given that each student goes through a series of emotions in his or her interaction with AutoTutor, can we automatically classify affect states from intelligent teaching systems to aid in the detection of a learner’s emotional state? Using frequency patterns of AutoTutor feedback and assigned user emotion in series of pairs, can the next pair of feedback/emotion series be predicted? This work will apply several machine learning classifiers to automatically identify affect states and select the best classifier. Additionally, we will apply a priori data mining to feedback/emotion series to determine if the next series can be predicted.

## 2. Materials and Methods

The Tutoring Research Group (TRG) at the University of Memphis has developed AutoTutor, which simulates human tutors and converses with students in natural language. Autotutor is a simulated human tutor program that has the capability of interacting with a student in a natural language. Utilizing a curriculum script, the Autotutor engages the student using mixed-initiative dialogue by presenting a set of problems. As the student constructs a correct answer to the problem, Autotutor, will present a series of prompts, hints, pumps (e.g., tell me more) to aid the student toward an answer. With additional dialogue, a full answer to a question is obtained within an average of 30 or 200 turns between the student and the tutor. Participants were videotaped during their AutoTutor sessions. They were asked to make verbal reports when they experienced one of the following affective states: anger, boredom, confusion, contempt, curiosity, disgust, delight, and frustration.

A session with AutoTutor is comprised of a set of questions that cover specific areas of the hardware, Internet, and operating systems. As described above, each subtopic is manifested by a series of turns in which AutoTutor maintains a conversation with the student in an attempt to construct an answer to the current subtopic. When an acceptable answer with the appropriate details is gleaned from the student’s responses, AutoTutor moves to the next subtopic. The participants were tutored with AutoTutor on one of three major computer literacy topics: hardware, operating systems, or the internet.

At the end of each student turn, AutoTutor captures a log file containing the student’s response, a variety of assessments of the response, and the tutor’s next move. The information can be divided into five categories: session information, response information, latent semantic analysis (LSA) assessments, the dialog advancer, and the tutor’s feedback.

The session information can be interpreted as a combination of global and local temporal markers that span across the period of interaction. The subtopic number indicates the number of questions answered. The turn provides a local measure of the number of student responses directed toward the current question. Response information considered was the verbosity of the response. The verbosity is considered to be the number of words and characters in the student’s response.

Local assessments for a given turn measure the student’s response for that turn on the basis of its similarity to good and bad answers. The Local Good Score is the highest match to the set of expectations representing good answers. The Local Bad Score is the highest match to the set of bad answers. A high Local Good Score is indicative of progress, while a high Local Bad Score can be interpreted as a student’s misconception. The Delta Local Good Score and the Delta Local Bad Score measure changes in the Local Good Score and the Local Bad Score, respectively. At the end of each student turn, a dialog move is chosen. The move chosen can be regarded as an indicator of the amount of information revealed to the student.

Dialog moves can be mapped onto a scale in the order: pump, hint, prompt, assertion, and summary. A pump conveys a minimum amount of information to the student and a summary conveys the most amount of explicit information. AutoTutor’s feedback is manifested in its verbal content, intonation, and other non-verbal conversational cues. Feedback consists of variations of negative, positive, and neutral responses.

### 2.1. Classification of Answers to Emotions

**Figure 1:**
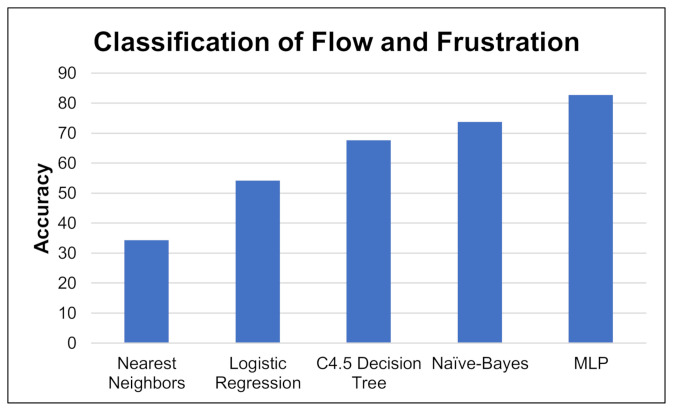
Performance of classification approaches *using* emotions ‘Flow’ and ‘Frustration’. MLP—Multilayer Perceptron.

The Waikato Environment for Knowledge Analysis (WEKA) analysis tool [19], was used to evaluate several classifiers in order to classify affects for each emotion from dialogue. Classifiers that were investigated were logistic regression, nearest neighbor, decision tree classifiers, naive Bayes and Multilayer Perceptron (MLP). Applying the classifiers across the emotions with the highest accuracy through pre-processing, ‘Flow’ and ‘Frustration’, we selected the classifier with the highest accuracy, See Figure 1. We created data models for the remaining classifiers that had the highest accuracy in order to further improve the overall accuracy of classifier, See Table 1.

The naive Bayes technique estimates class-conditional probability using an assumption of conditional independence among attributes given a class label. The attributes are the affect states that preceded next emotion. Conditional independence is described as follows: If X, Y, and Z are three sets of random variables, then the variables in X are conditionally independent of Y, given Z, if P(X|Y, Z) = P(X|Z). Using this conditional independence assumption, the classifier estimates the conditional probability of each X, given Y. This approach is desirable in that it does not require a large training set to obtain a good estimate of probability [20].

With MLP, an Artificial Neural Network technique, there is a layer of input nodes, one for each attribute and a layer of output nodes, one for each output class. One or more layers may exist in between. For each node, each of the inputs has a weight assigned. All of the inputs to a node are weighted and summed. The result is normalized to a value between 0 and 1 or −1 and 1. The result of each layer becomes input to the next layer. Using an iterative back-propagation algorithm with the training data, MLP adjusts weights that contribute to misclassifications. The iterative process continues until the error rate is adjusted to an acceptable level.

**Figure 2:**
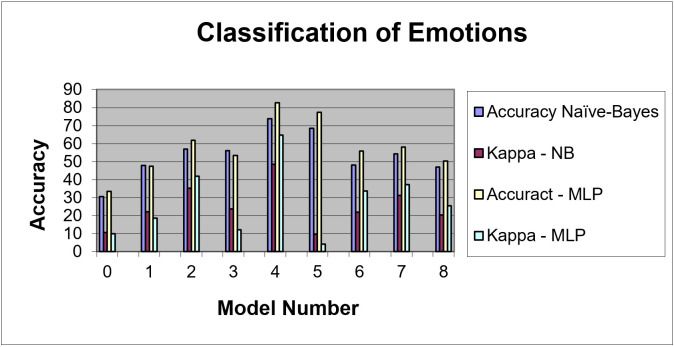
Classification of Emotions. NB—naive Bayes.

The Cohen’s Kappa statistic was used to assess inter-rater reliability when observing qualitative or categorical variables. Kappa is considered to be an improvement over using percent accuracy to evaluate this type of reliability. Kappa has a range from 0–1.00. with larger values indicating better reliability. Generally, a Kappa greater than 0.70 is considered satisfactory. Performance of the remaining classifiers is on Figure 2.

### 2.2. Prediction of a Next Series of Emotions

Preceding values of feedback and emotions are used to predict the next series of feedback and emotions. The goal of this predictive modeling is to model the history of data in order to accurately forecast future unknown data values. A three- and five-layer MLP network is trained to predict the next value of the time series using preceding values as input. A generalization of naive Bayes is used to build a Bayesian network for predictive modeling in Weka.

### 2.3. Discovering Interesting Relationships in the AutoTutor Data

Mining for associations among items in a large database is an important data mining function. An implementation of the a priori algorithm is used to mine frequent item sets, also known as association rules. Support is defined as the number of transactions that contain a particular item set. If an item set satisfies a minimum support threshold, then it is a frequent item set. An item set satisfies minimum support if the occurrence frequency of the item set is greater than or equal to the user supplied threshold. In the first step of the algorithm, the set of frequent 1-item sets if found. This set is used to find the set of frequent 2-itemsets, which is then used to find frequent 3-item sets. This process is repeated until no more frequent k-item sets can be found.

Confidence measures the reliability of the inference made by a rule. Once the frequent item sets are identified the rules with high confidence are extracted. The higher the confidence of a rule, the more likely that the consequence will be present in transactions that contain the antecedent. An association rule suggests a strong co-occurrence relationship between items in the antecedent and consequent of the rule [20].

### 2.4. Participants

Thirty-four college students provided 200 turns between each student and the AutoTutor. The participants were given the list of affective states, i.e., (1) anger, (2) boredom, (3) confusion, (4) contempt, (5) curiosity, (6) delight, (7) flow, and (8) frustration. They were instructed they would be expressing their affective states while learning about computer literacy with a computer system called AutoTutor by emoting aloud each affective state [21]. Five trained judges recorded emotions that learners were apparently experiencing every 5 min during the 90 min of interaction with AutoTutor on topics in computer literacy (e.g., hardware, internet, or operating systems). A video of the participant’s face was captured using the IBM blue-eyes camera. A screen capturing software program called Camtasia Studio was used to capture the audio and video of the participant’s entire tutoring session. Judges were also instructed to make judgments on what affective states were present at 20-s intervals; at each of these points, the video automatically paused (freeze-framed). Judges were also instructed to indicate any affective states that were present in between the 20-s stops. The five judges would apply a score from the Facial Action Coding System [22] onto the screen capture of the participants from the multiple affects states. The coding from judges was placed into a database with a timestamp. There were roughly two facial scores for every emote aloud task.

### 2.5. Data Preparation

Affect data were captured into log files and reviewed before processing. Data in the log files for several rows were missing, due to either data sensor device driver or human interaction mishap. This issue caused data analysis for several participants to be dropped. Additionally, data collected from emote-aloud feedback had extremely low instances of contempt, curiosity, and disgust, so these affective states were not included in the current analysis. Anger and eureka were exhibited in very few participants and were also excluded. Reliable data that were captured for most of the cohorts was boredom confusion, flow, and frustration. Boredom and confusion were the most frequent affective states in the Craig et al. (2004) study that had 34 participants [15]. Therefore, we will use four sets of participants’ data in the current study as representation of a larger population.

## 3. Results

### 3.1. Classification of Answers to Emotions

Using naive Bayes and MLP, classifications were performed with varying sets of emotions as shown in Table 1. Results from each were analyzed and compared, and a summary of the comparison is given below.

Overall, the MLP technique classified the attributes better than naive Bayes (82.75% vs. 73.79%). The best classification occurred with the relative probabilities of the attributes for emotions ‘Flow’ and ‘Frustration’. These two emotions represent the furthest ends of both sides of the emotion spectrum for AutoTutor, therefore it would be expected that their attributes would be the most stratified, as shown in Figure 2. Accuracy naive Bayes vs. Kappa-NB datasets involve the selection of data to process without a Kappa measure vs. processing data that has a Kappa measure. Accuract MLP vs. Kappa-MLP datasets involves the selection of data to process without a Kappa measure vs. processing data that has a Kappa measure.

Improvement of classification accuracy is displayed when we remove all attributes corresponding to an emotion except ‘Flow’ and ‘Frustration’. A series of tests are then performed to determine if the emotions ‘Flow’ and ‘Frustration’ are the better classified because they are compared against each other, or if their attributes truly correspond to their respective class emotions.

The emotion classes, ‘Flow’ and ‘Frustration’ are classified against the entire set of emotions, except all of the emotions are set to the class label of ‘Neither’. Due to the great number of ‘Neither’ classes compared to the classes ‘Flow’ and ‘Frustration’, a ratio of 6:1, the accuracy of classification is still high (Model 5), but the Cohen’s Kappa had shrunk so dramatically, that if you had to guess whether a series of attributes was ‘Flow’, ‘Frustration’ or ‘Neither’, almost all the time you would guess ‘Neither’.

Fifty rows each of ‘Neither’, ‘Flow’, and ‘Frustration’ were identified and used in the classification. Accuracy was down slightly, but the Cohen’s Kappa improved. The set representing ‘Neither’ was changed from random emotions to two labeled emotions, ‘Neutral’ and ‘Delight’. Accuracy remained consistent at approximately 60%, and the Cohen’s Kappa improved to roughly ~40% in Model 7.

### 3.2. Prediction of a Next Series of Emotions

Two series of attributes and emotions were concatenated into one row to create a record of previous and next set of emotions. If these series of attributes and emotions could be classified accurately, then it would be possible to predict a series of emotions based on previous classification of attributes and emotions. Table 2 lists the algorithms used for classification with accuracy and Cohen’s Kappa measures.

The naive Bayes approach together with MLP provided low accuracy and Kappa values. Increasing the amount of weights and training time, from 0.3 to 0.5 and 500 to 1000, respectively, did not improve the overall accuracy of classifying the series of attributes and emotions.

The next section discusses an approach using six sequences of attributes and emotion, (frustration, confusion, neutral, delight, boredom, flow) where the attributes uses Auto Tutor feedback (positive, negative, neutral-positive, neutral-negative) to the participant. The user’s emotions are assigned to Auto Tutor feedback and are used in the next section for analysis.

### 3.3. Discovering Interesting Relationships in the AutoTutor Data

Test sets were gathered from Auto Tutor participants to find Frequent Item Sequences, sequences with frequency higher than 10%, similar subsets within a set of data, and similar Frequent Item Sequences between data sets. Test sets with Frequent Item Sequences below 10% were discarded for this experiment.

For each data set, the following relationships are identified:

- Frequent Item sets that have high percentages of instances of particular emotions and the applied feedback-based stimulation (FBS) from Auto Tutor that preceded the series of emotions, i.e., positive, negative or neutral feedback.
- Similar sequences for this participant that display a sequence of emotions.
- Antecedent/Consequent feedback/emotions that display the next set of feedback and emotions derived from similar sequences found in the previous step.

In addition to these relationships, matches between two groups and between all groups are identified.

For participant 36 and 44, 37.5% instances have the participant displaying emotions from Confusion, Neutral, to Frustration based on negative feedback from AutoTutor as shown in Table 3.

For Participant 43 in Table 4, 30% or more instances have emotions of Neutral to Flow based on negative and positive feedback from Auto Tutor. Participant 45 has 30% or more instances have emotions of Confusion to Neutral based on negative feedback from Auto Tutor.

Regarding Participant 36, there are slightly more instances that start from Confusion to Neutral and then back to Confusion as revealed in Table 5 as opposed to other frequent item sets found in Table 5, based on positive, then negative and then negative feedback. Similar sequences for Participant 44 start from Confusion to Neutral and then Neutral/Flow by flipping the feedback to the student. Regarding participant 43, more than 18% of the occurrences found similar sequences begin at the Boredom affective state when positive feedback is applied, then to Neutral when positive feedback is applied, and then Flow when positive feedback is applied again. Additionally, similar sequences start from Neutral when positive feedback is applied, then to Delight when negative feedback is applied, and then Neutral when negative feedback is applied again. Participant 45 found 18% similar sequences start from Neutral to Delight and then back to the Neutral affective state based on positive, negative and then negative feedback.

For participant 36, the following similar sequences that match previously identified frequent item sets were found:

- positive-feedback-FBS-Delight positive-feedback-FBS-Confusion negative-feedback-FBS-Neutral with support of 12.5%.
- positive-feedback-FBS-Delight positive-feedback-FBS-Confusion negative-feedback-FBS-Confusion with support of 12.5%.

Antecedent and consequent are found in the following similar subsequences. Positive feedback followed by negative feedback changes the emotion of the participant from Delight to Neutral/Confusion, with the next subsequence most likely being Confusion/Neutral.

- positive-feedback-FBS-Delight positive-feedback-FBS-Confusion negative-feedback-FBS-Neutral leads to negative-feedback-FBS-Confusion with support of 12.5% and 100.0% confidence.
- positive-feedback-FBS-Delight positive-feedback-FBS-Confusion negative-feedback-FBS-Confusion leads to negative-feedback-FBS-Neutral with support of 12.5% and 100.0% confidence.

Similar sequences for Participant 44 start from Confusion to Neutral and then Neutral/Flow. These frequent item Sequences lead to the following antecedent and consequent. Negative feedback followed by positive feedback changes the emotion of the participant from Confusion to Neutral, where the next subsequence will probably be Flow/Neutral, respectively:

- Neutral-negative-feedback-FBS-Confusion positive-feedback-FBS-Neutral leads to positive-feedback-FBS-Flow with support of 12.5% and 100.0% confidence.
- Negative-feedback-FBS-Confusion neutral-negative-feedback-FBS-Neutral leads to negative-feedback-FBS-Neutral with support of 12.5% and 100.0% confidence.

For participant 43, antecedent and consequent are found in the following similar subsequences. When positive and neutral negative feedback is applied to Confusion/Flow emotions, respectively and then positive feedback is applied to Neutral emotions, the next series of emotions is Flow. Improvement is measured when ‘positive feedback’ from AutoTutor is applied.

- Positive-FBS-Boredom positive-FBS-Neutral leads to positive-FBS-Flow with support of 18.8% and 100.0% confidence.
- Neutral-negative-feedback-FBS-Flow/Confusion positive-FBS-Neutral leads to positive-FBS-Flow with support of 12.5% and 100.0% confidence.

For participant 45, the following similar sequences that match previously identified frequent item sets were found:

- Positive-feedback-FBS-Neutral negative-feedback-FBS-Delight leads to negative-feedback-FBS-Neutral with support of 12.5% and 100.0% confidence.
- Positive-feedback-FBS-Delight negative-feedback-FBS-Confusion leads to negative-feedback-FBS-Neutral with support of 12.5% and 100.0% confidence.

Antecedent and consequent are found in the following similar subsequences. Positive/Negative feedback followed by negative feedback changes the emotion of the participant from Neutral/Delight to Confusion to Neutral.

Similar sequences between groups show that each participant in this study share the same emotions when a sequence of feedback prompts is applied from AutoTutor. Selection is based on two out of three subsequences where the maximum number of similar sequences are found across the participants. Similar Sequences between Participants 43 and 45 are revealed in Table 6. Between these two participants, positive feedback applied to Neutral emotions, and then positive/negative feedback applied to Flow/Delight emotions also has final emotions that are Confusion.

Table 7 displays similar sequences between participants when positive and negative feedback are initially applied when the initial affective state is Delight. Similar outcomes are derived when positive and negative feedback are continually applied. A Neutral outcome is the final state when negative feedback is applied.

Table 8 displays an opposite outcome when the initial affective state is Confusion and either positive and negative feedback are initially applied. When positive feedback is continually applied the final affective state Flow.

Table 9 shows similar sequences between Participants 36, 44 and 45. Looking at Participants 44 and 36 within this group, negative feedback applied to Confusion emotions, and then negative feedback applied to Neutral emotions also has emotions that are Neutral/Frustration. Positive feedback applied to Confusion emotions, and then negative feedback applied to Neutral emotions also has emotions that are either Delight/Confusion.

## 4. Discussion

Throughout this study, it has been observed how feedback can influence affective states of the participant during a learning session. In some instances, and depending on the type of feedback, participants have changed their affective state from Boredom/Confusion to Neutral and finally to Flow. Confusion and Flow were positively associated with learning gains but Boredom was negatively associated with learning [16]. Lee and colleagues [23] have found that content and duration of affect matters with Boredom and Confusion correlated positively to learning sessions in the short term, but turning negative in the after longer sessions. Conversely, when the participant is in an affected state of Flow, longer term sessions can involve positive engagement in a specific domain [24].

Single participant observation’s affective states are captured through video capture via participant’s emote aloud approach [3], where participants report their current emotional state during a learning session. Two, three, and four frequent item states begin to emerge that show unique rows of affective states. We also see from Table 3 and Table 4, higher incidents of affective states per participants after feedback has been applied:

- Participant 36: Confusion: 68.8%.
- Participant 43: Flow: 62.5%.
- Participant 44: Confusion: 68.8%.
- Participant 45: Confusion: 62.5%.

The learner is aware of their emotional state as they are progressing through a particular lesson plan, such as learning about an operating system. It is possible that the learner becomes self-aware of their current emotional state and regulates their emotional range of emotions to Confusion, Frustration, and Boredom to represent their negative emotional state, and selects Flow and Delight on the positive side to limit their emotions through an affective state spectrum [20]. The neutral state represents either a reset affective state or an emotional state that the participant chooses not to express at a given instance.

Similar sequences for a participant involve the application of three feedback sequences to emotive responses. Confusion, Flow and Neutral were the most common outcomes for each sequence, with Confusion and Flow having the highest incidence at 18.8%, as seen in Table 5. Depending on the participant, we see how positive or negative feedback to positive affective states such as Delight can eventually lead to an affective state of Confusion. Similarly, we see how positive or negative feedback to negative affective states such as Confusion can eventually lead to an affective state of Flow. Initial applications of positive or negative feedback to Neutral states leads to the affective Neutral state. Through a priori analysis, oscillatory behavior was found due to the presence of positive to negative and negative to positive affective states. As mentioned in the introduction, when the student feels Confusion, Autotutor prompts the student to enter into a learning state of curiousness and eventually, ‘Flow’. After a while, the Flow state may turn back into Confusion assuming new or even more challenging material is presented to the student. Even the student may mistake the Confusion affective state for the state of Curiousness, enabling moments of insight during problem solving sessions [15], once again causing the student to further engage with Autotutor. Additionally, Confusion has been found in previous research as the impetus behind the emotion to problem solving and deep thinking [17,25,26,27,28,29], causing the cycle of Confusion->Neutral->Flow.

Similar sequences between participants display shared sequences in order to determine if the application of positive/negative feedback can derive similar affective states. Participants 43 and 45 have the same initial and final affective state, yet the application of positive/negative feedback is different. There is a slightly higher incident in one feedback route over the other that will provide the desired outcome (18.8% vs. 12.5%) displayed in Table 6. Participants 36 and 45 share similar affective states Delight, Confusion and finally Neutral, seen in Table 7. Additionally, participants 43 and 44 share similar affective states Confusion, Neutral and finally Flow, seen in Table 8. Finally, the initial application of either positive or negative feedback yields a Confusion affective state, displayed in Table 9. Negative feedback is then applied across all participants. Negative feedback is applied once again, yielding various responses per participant, with the participant having the highest incident of 37.5% of the affective state Frustration.

The high incidence of Confusion as stated previously may be due to the participant’s problem-solving process. We would see Confusion in almost every instance due to the fact they are processing a new problem [16,17]. The other aspect of Confusion is the negative state where AutoTutor cannot help the participant find a solution and the participant becomes further frustrated, either through design of the lesson or additional support needed for the participant. This leads to the cause of high instances Frustration. Regarding Table 3, when Frustration and Confusion are present such as for Participant 36 and 44, they become the dominant affective states at 62.5% and 68.5%, respectively, as opposed to Table 4, where Flow is present and becomes the dominant affective states for Participant 43 at 62.5%. Previous research has found that frustrated students are more intractably to stay frustrated, while happy students, i.e., students who exhibit affective states of Flow/Delight, can easily transition into other affective states [7].

## 5. Conclusions

The objectives that were set out for this research were: Given that each student goes through a series of emotions in his or her interaction with AutoTutor, can we automatically classify affect states from intelligent teaching systems to aid in the detection of a learner’s emotional state? Using frequency patterns of AutoTutor feedback and assigned user emotion in series of pairs, can the next pair of feedback/emotion series be predicted? This second objective seems to compliment the major goal of the ITSPOKE project at the University of Pittsburgh, to obtain an understanding of whether cues available to spoken dialogue systems can be used to predict emotion and ultimately to improve tutoring performance [24]. In order to identify any interesting associations among the participant interaction data, frequent item sets were identified. Item sets with length less than three were discarded along with item sets with low support and confidence. For frequency sets consisting of a single row of interaction log information, low support and low confidence were identified. With an average AutoTutor session consisting of six feedback rows, ten sequences in a row were considered. Again, support and confidence measures were low.

While prediction of next series of emotions proved unattainable in this exercise, the following determinations against the stated project objectives were made:

- The emotions ‘Flow’ and ‘Frustration’ had the highest classification of all the other emotions when measured against other emotion attributes and different distinct sets.
- A set of common rules were extracted per participant and between participants based on Auto Tutor feedback and user’s emotions. The most common frequent item sets were ‘Flow’ and ‘Confusion’.

MLP technique classified the attributes better than naive Bayes (82.75% vs. 73.79%) for Flow and Frustration. Having the capability to accurately distinguish a series of emotions will enable not only educators better interact with learners in during teaching session, but can infuse automated teaching systems to look for these affects, and provide a learning session that is more attuned to the needs of the student. One of the more interesting findings seen in Table 5, through a priori analysis, was a type of oscillatory behavior that exhibited the presence of positive to negative and negative to positive affective states. As mentioned in the introduction, when the student feels Confusion, Autotutor prompts the student to enter into a learning state of curiousness and eventually, ‘Flow’. It seems possible to guide the student through an automated learning system toward a beneficial learning state.

As mentioned earlier, additional sensors have been employed beyond this study which involved body posture measurement and facial features [26]. Between our results which provide an automated classification through naive Bayes (82.75%) and a rapid human classification (90%) where both approaches provided a classification of affect states, machine learning classification is on its way to meet the needs for the online learning population.

Future work incorporates machine learning classification of affect states in real-time and adapts to the student’s emotional state in order to better facilitate a learning session. The automatic detection of affect states provides an empathetic level of intelligent learning systems, where artificial intelligence can not only provide a system to optimally provide content to the learner, but also provide educators a learning system to guide them in the affect states that occur during a learning process. Multi-modal sensors have been implemented in past, and there is still an opportunity to spatially align these systems, such as optimally pick up facial cues, look for physiological stressors either caused by the lesson or through external events, and finally provide the type of interface that enables a ’flow’ of information to occur. Through these efforts, automatic detection of a student’s affective states will become more feasible.

## Figures and Tables

**Table 1:** Sets of Emotions Used in Classification. MLP—Multilayer Perceptron.

Model	Algorithm	Emotions
0	naive Bayes	Keep All
	MLP	
1	naive Bayes	Remove Delight, Neutral, Surprise
	MLP	
2	naive Bayes	Remove Confusion, Delight, Neutral, Surprise
	MLP	
3	naive Bayes	Remove Boredom, Delight, Neutral, Surprise
	MLP	
4	naive Bayes	Remove all but Flow and Frustration
	MLP	
5	naive Bayes	Change all to ‘Neither’ but Flow and Frustration
	MLP	
6	naive Bayes	50 rows of ‘Neither’, 50 rows of Flow and Frustration
	MLP	
7	naive Bayes	50 rows of ‘Neutral’, 50 rows of Flow and Frustration
	MLP	
8	naive Bayes	50 rows of ‘Delight’, 50 rows of Flow and Frustration
	MLP	

**Table 2:** Classification comparison.

Algorithm	Accuracy	Kappa
naive Bayes	39.1379	0.235
MLP-500 (0.3)	38.6207	0.1983
MLP-1000 (0.5)	40	0.2167

**Table 3:** Participants 36 and 44: Frequent Item Sequences. (FBS—feedback-based stimulation)

Participant	Frequent Item Sets				% Instances
36					
1	positive-feedback-FBS-Confusion				31.3%
2	negative-feedback-FBS-Frustration				62.5%
3	negative-feedback-FBS-Neutral				62.5%
4	negative-feedback-FBS-Confusion				68.8%
5	negative-feedback-FBS-Frustration	negative-feedback-FBS-Neutral			50.0%
6	negative-feedback-FBS-Frustration	negative-feedback-FBS-Confusion			50.0%
7	negative-feedback-FBS-Neutral	negative-feedback-FBS-Confusion			50.0%
8	negative-feedback-FBS-Frustration	negative-feedback-FBS-Neutral	negative-feedback-FBS-Confusion		37.5%
9	positive-feedback-FBS-Delight	positive-feedback-FBS-Confusion	negative-feedback-FBS-Neutral	negative-feedback-FBS-Confusion	12.5%
44					
1	positive-feedback-FBS-Confusion				31.3%
2	negative-feedback-FBS-Frustration				62.5%
3	negative-feedback-FBS-Neutral				62.5%
4	negative-feedback-FBS-Confusion				68.8%
5	negative-feedback-FBS-Frustration	negative-feedback-FBS-Neutral			50.0%
6	negative-feedback-FBS-Frustration	negative-feedback-FBS-Confusion			50.0%
7	negative-feedback-FBS-Neutral	negative-feedback-FBS-Confusion			50.0%
8	negative-feedback-FBS-Frustration	negative-feedback-FBS-Neutral	negative-feedback-FBS-Confusion		37.5%
9	positive-feedback-FBS-Delight	positive-feedback-FBS-Confusion	negative-feedback-FBS-Neutral	negative-feedback-FBS-Confusion	12.5%

**Table 4:** Participants 43 and 45: Frequent Item Sequences.

Participant	Frequent Item Sets				% Instances
43					
1	neutral-negative-feedback-FBS-Flow				31.30%
2	positive-feedback-FBS-Neutral				37.50%
3	negative-feedback-FBS-Neutral				43.80%
4	positive-feedback-FBS-Flow				62.50%
5	negative-feedback-FBS-Flow				62.50%
6	negative-feedback-FBS-Neutral	negative-feedback-FBS-Flow			31.30%
7	positive-feedback-FBS-Neutral	positive-feedback-FBS-Neutral	positive-feedback-FBS-Confusion	negative-feedback-FBS-Neutral	12.50%
45					
1	positive-feedback-FBS-Neutral				31.30%
2	negative-feedback-FBS-Delight				37.50%
3	negative-feedback-FBS-Confusion				37.50%
4	negative-feedback-FBS-Neutral				62.50%
5	negative-feedback-FBS-Delight	positive-feedback-FBS-Confusion			56.30%
6	negative-feedback-FBS-Delight	negative-feedback-FBS-Neutral			25.00%
7	positive-feedback-FBS-Neutral	positive-feedback-FBS-Confusion			25.00%
8	positive-feedback-FBS-Confusion	negative-feedback-FBS-Neutral			25.00%
9	negative-feedback-FBS-Confusion	negative-feedback-FBS-Neutral			31.30%
10	positive-feedback-FBS-Neutral	negative-feedback-FBS-Delight	positive-feedback-FBS-Confusion	negative-feedback-FBS-Neutral	12.50%

**Table 5:** Similar Sequences.

Participant	Frequent Item Sets			% Instances
**36**				
**1**	positive-feedback-FBS-Delight	positive-feedback-FBS-Confusion	negative-feedback-FBS-Confusion	12.5%
**2**	positive-feedback-FBS-Delight	positive-feedback-FBS-Confusion	negative-feedback-FBS-Neutral	12.5%
**3**	positive-feedback-FBS-Delight	negative-feedback-FBS-Neutral	negative-feedback-FBS-Confusion	12.5%
**4**	negative-feedback-FBS-Delight	negative-feedback-FBS-Neutral	negative-feedback-FBS-Confusion	12.5%
**5**	positive-feedback-FBS-Confusion	negative-feedback-FBS-Neutral	negative-feedback-FBS-Confusion	18.8%
**6**	negative-feedback-FBS-Delight	negative-feedback-FBS-Frustration	negative-feedback-FBS-Neutral	12.5%
**7**	negative-feedback-FBS-Delight	negative-feedback-FBS-Frustration	negative-feedback-FBS-Confusion	12.5%
**43**				
**1**	positive-feedback-FBS-Confusion	positive-feedback-FBS-Neutral	positive-feedback-FBS-Flow	12.5%
2	neutral-negative-feedback-FBS-Flow	positive-feedback-FBS-Neutral	positive-feedback-FBS-Flow	12.5%
3	positive-feedback-FBS-Boredom	positive-feedback-FBS-Neutral	positive-feedback-FBS-Flow	18.8%
**44**				
**1**	neutral-negative-feedback-FBS-Confusion	positive-feedback-FBS-Neutral	positive-feedback-FBS-Flow	12.5%
**2**	negative-feedback-FBS-Confusion	neutral-negative-feedback-FBS-Neutral	negative-feedback-FBS-Neutral	12.5%
**45**				
**1**	positive-feedback-FBS-Neutral	negative-feedback-FBS-Delight	positive-feedback-FBS-Confusion	18.8%
**2**	positive-feedback-FBS-Neutral	negative-feedback-FBS-Delight	negative-feedback-FBS-Neutral	12.5%
**3**	negative-feedback-FBS-Delight	positive-feedback-FBS-Confusion	negative-feedback-FBS-Neutral	12.5%
**4**	positive-feedback-FBS-Delight	negative-feedback-FBS-Confusion	negative-feedback-FBS-Neutral	12.5%

**Table 6:** Similar Sequences between 43 and 45.

Participant	Frequent Item Sets			% Instances
**43**	positive-FBS-Neutral	positive-feedback-FBS-Flow	positive-FBS-Confusion	12.5%
**45**	positive-FBS-Neutral	negative-feedback-FBS-Delight	positive-FBS-Confusion	18.8%

**Table 7:** Similar Sequences between 36 and 45.

Participant	Frequent Item Sets			% Instances
**36**	positive-feedback-FBS-Delight	positive-feedback-FBS-Confusion	negative-feedback-FBS-Neutral	12.5%
**45**	negative-feedback-FBS-Delight	positive-feedback-FBS-Confusion	negative-feedback-FBS-Neutral	12.5%
**45**	positive-feedback-FBS-Delight	negative-feedback-FBS-Confusion	negative-feedback-FBS-Neutral	12.5%

**Table 8:** Similar Sequences between 43 and 44.

Participant	Frequent Item Sets			% Instances
**43**	positive-feedback-FBS-Confusion	positive-feedback-FBS-Neutral	positive-feedback-FBS-Flow	12.5%
**44**	neutral-negative-feedback-FBS-Confusion	positive-feedback-FBS-Neutral	positive-feedback-FBS-Flow	12.5%

**Table 9:** Similar Sequences between 36, 44 and 45.

Participant	Frequent Item Sets			% Instances
**36**	negative-feedback-FBS-Confusion	negative-feedback-FBS-Neutral	negative-feedback-FBS-Frustration	37.5%
**43**	positive-feedback-FBS-Confusion	negative-feedback-FBS-Neutral	negative-feedback-FBS-Confusion	18.8%
**44**	negative-feedback-FBS-Confusion	negative-feedback-FBS-Neutral	neutral-negative-feedback-FBS-Neutral	12.5%
**45**	positive-feedback-FBS-Confusion	negative-feedback-FBS-Neutral	negative-feedback-FBS-Delight	12.5%

## Data Availability

Data is available upon request.
